# A case of post COVID‐19 multisystem inflammatory syndrome and Bell's palsy in a young adult

**DOI:** 10.1002/ccr3.4801

**Published:** 2021-09-09

**Authors:** Abdulrahman F. Al‐Mashdali, Musaed S. Al Samawi

**Affiliations:** ^1^ Department of Internal Medicine Hamad Medical Corporation Doha Qatar; ^2^ Department of Infectious Diseases Hamad Medical Corporation Doha Qatar

**Keywords:** Bell’s palsy, COVID‐19, MIS‐A, multisystem inflammatory syndrome

## Abstract

The clinician should put MIS‐A at the top of differential diagnoses of a patient with febrile illness and multiple organ dysfunction during the early post‐COVID‐19 period. Also, facial nerve palsy might follow COVID‐19, related to the autoimmune phenomenon.

## Graphical abstract

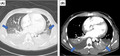

## 1 BACKGROUND

The clinician should put MIS‐A at the top of differential diagnoses of a patient with febrile illness and multiple organ dysfunction during the early post‐COVID‐19 period. Facial nerve palsy is one of the COVID‐19‐related neurological complications that might occur within few weeks after the disease.

COVID‐19 is a respiratory illness caused by severe acute respiratory syndrome coronavirus 2 (SARS‐CoV‐2), a new strain of coronavirus discovered in 2019. Multisystem inflammatory syndrome in children (MIS‐C) is an uncommon systemic disease that followed a recent COVID‐19 infection in children and adolescents. The primary presenting manifestation of this syndrome is fever, which presents in all cases, and multisystem involvement, which mainly affects cardiovascular and gastrointestinal systems.^1^

A similar condition has recently been identified in adults, known as a multisystem inflammatory syndrome in adults (MIS‐A).^2^ However, MIS‐A cases have been rarely encountered during the COVID‐19 era; in a recent review, only 51 cases have been reported in patients ≥18‐year‐old since the beginning of the COVID‐19 outbreak, and thus, the current evidence for the management of MIS‐A is still scarce.^3^

Herein, we report a rare and interesting case of a 21‐year‐old man who presented three weeks after mild COVID‐19 with the typical features for MIS and developed unilateral Bell^’^s palsy after that. In this case, we highlight the importance of considering MIS‐A in adult patients who present within a few weeks after COVID‐19 infection with unremitting fever and manifestations of multiorgan dysfunction.

## 2 CASE PRESENTATION

A 21‐year‐old man with no past medical history of chronic illnesses presented to the emergency department (ED) with fever and abdominal pain for one week. Three weeks before this presentation, the patient was admitted to the hospital with fever and cough; after that, he was found to have a COVID‐19 infection. He was discharged with antipyretic for home isolation, and his symptoms settled within few days. However, two weeks after the diagnosis with COVID‐19, he again came with fever and watery diarrhea. At that time, repeated RT‐PCR for SARS‐CoV2 was negative with positive serology. He was discharged home on an oral antibiotic.

The patient presented with continuous fever at the latest presentation, although he had completed five days of antibiotic. The patient also complained of generalized severe abdominal pain, vomiting, and diarrhea for one week. However, he denied any respiratory symptoms. On physical examination, the patient was unwell but alert and oriented to time, place, and person. His vital signs were significant for an oral temperature of 39°C, pulse rate of 110 beats per min, blood pressure of 115/70 mmHg, respiratory rate of 18 breaths/min, and oxygen saturation of 98% on room air. Abdominal examination revealed generalized mild tenderness without guarding or rigidity. In addition, an enlarged tender left cervical lymph node and bilateral non‐purulent conjunctivitis were noticed. Apart from previous findings, respiratory, cardiovascular, and neurological examinations were unremarkable.

COVID‐19 polymerase chain reaction (PCR) was negative with a positive serologic test that indicated the previous infection. Laboratory investigations were significant for a white blood cell count of 4.7 × 10^3/ul, a hemoglobin level of 11.5 gm/dl, a Platelet count of 111 × 10^3/ul, and c‐reactive protein of 155 mg/l; the relevant laboratory findings at the presentation are listed below in Table 1. Urinalysis showed microscopic hematuria and pyuria. The human immunodeficiency virus (HIV) screen and respiratory viral panel (using multiplex PCR‐based assays) were negative. Electrocardiography (ECG) and chest x‐ray (CXR), and abdominal ultrasonography did not show any significant findings. Neck ultrasound revealed multiple hypoechoic lymph nodes noted on the neck's left side, measuring 1.9 × 0.7 cm in size. Based on the previous clinical and laboratory findings, the patient was suspected of having sepsis and started on ceftriaxone empirically.

**TABLE 1:** Laboratory findings during the hospital course

Parameter	Day 1	Day 2	Day 7 (After 48 h of starting meropenem and linezolid)	Normal Range
WBC	4.7 × 10^3/ul	4.0 × 10^3/ul	7 × 10^3/ul	4.0‐10.0
Hgb	11.5 gm/dl	9.5 gm/dl	9.2 gm/dl	13.0‐17.0
Platelet	111 × 10^3/ul	79 × 10^3/ul	261 × 10^3/ul	150‐400
INR	N/A	1.8	1.2	
PT	N/A	17.8 s	12.8 s	9.7‐11.8
APTT	N/A	24.5 s	30.1 s	24.6‐31.2
D‐dimer	N/A	2.87 mg/dl	N/A	0‐0.46
Urea	3.6 mmol/L	4.3 mmol/L	4.2 mmol/L	2.5‐7.8
Creatinine	90 umol/L	114 umol/L	69 umol/L	62‐106
Sodium	136 mmol/L	135 mmol/L	137 mmol/L	133‐146
Potassium	3.8 mmol/L	3.5 mmol/L	4 mmol/L	3.5‐5.3
ALT	57 U/L	52 U/L	20 U/L	0‐41
AST	92 U/L	72 U/L	51 U/L	0‐40
Lipase	10 U/L	N/A	N/A	13‐60
CRP	156.4 mg/L	223 mg/L	58.2	0.0‐5.0
Procalcitonin	0.30 ng/ml	2.5 ng/ml	0.78 ng/ml	Below 0.5
NT‐proBNP	N/A	6,836 pg/ml	N/A	Below 125
Troponin‐T	N/A	78 ng/L	N/A	Below 15
Ferritin	N/A	1,023 ug/L	N/A	Below 300

Abbreviations: ALT, alanine aminotransferase; APTT, activated partial thromboplastin time; AST, aspartate aminotransferase; CRP, C‐reactive protein; Hgb, Hemoglobin; INR, International normalization ratio; N/A, Not available; NT‐proBNP, N‐terminal pro‐B‐type natriuretic peptide; PT, Prothrombin time; WBC, White blood cell.

**FIGURE 1:**
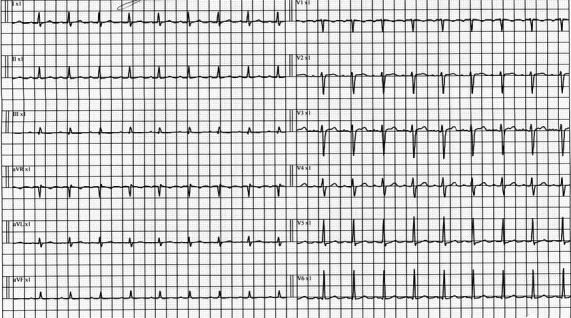
ECG on day 2 of hospital course reveals sinus tachycardia and low voltage QRS in limb leads

**FIGURE 2:**
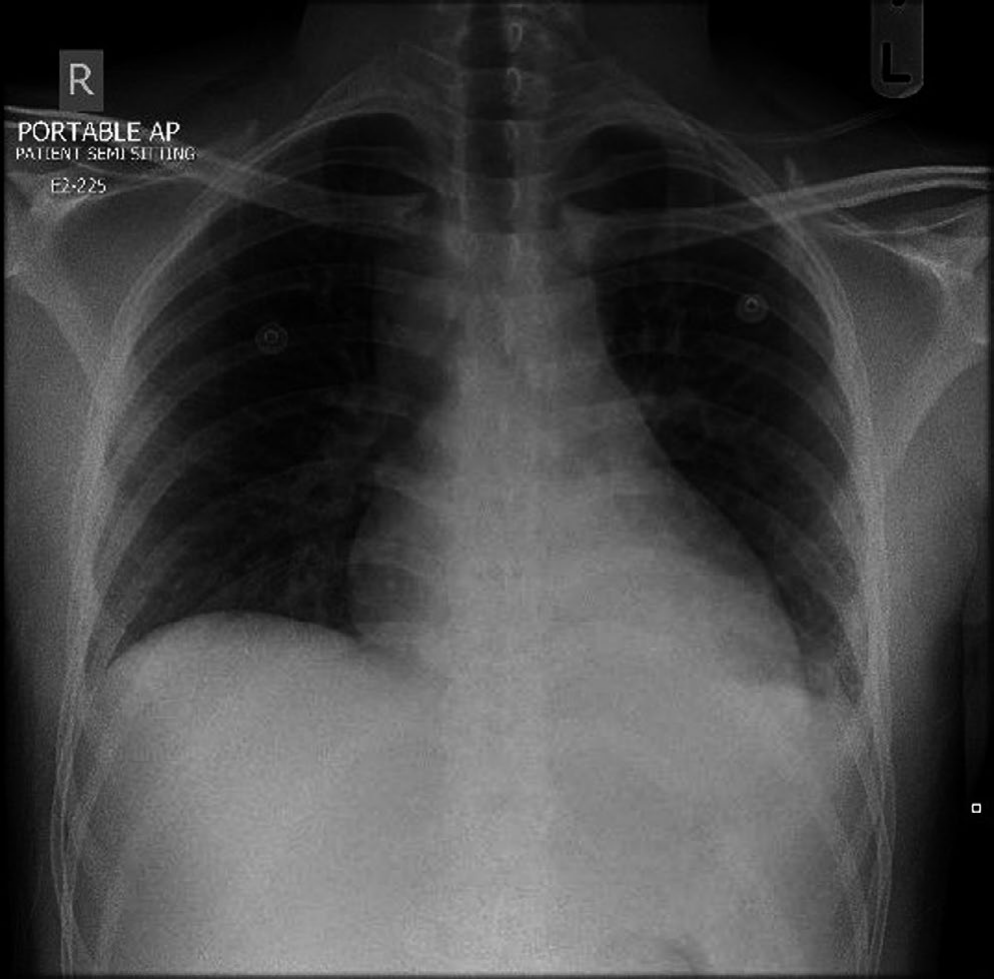
CXR shows a new hazy opacity in the left lower zone of the chest

**FIGURE 3:**
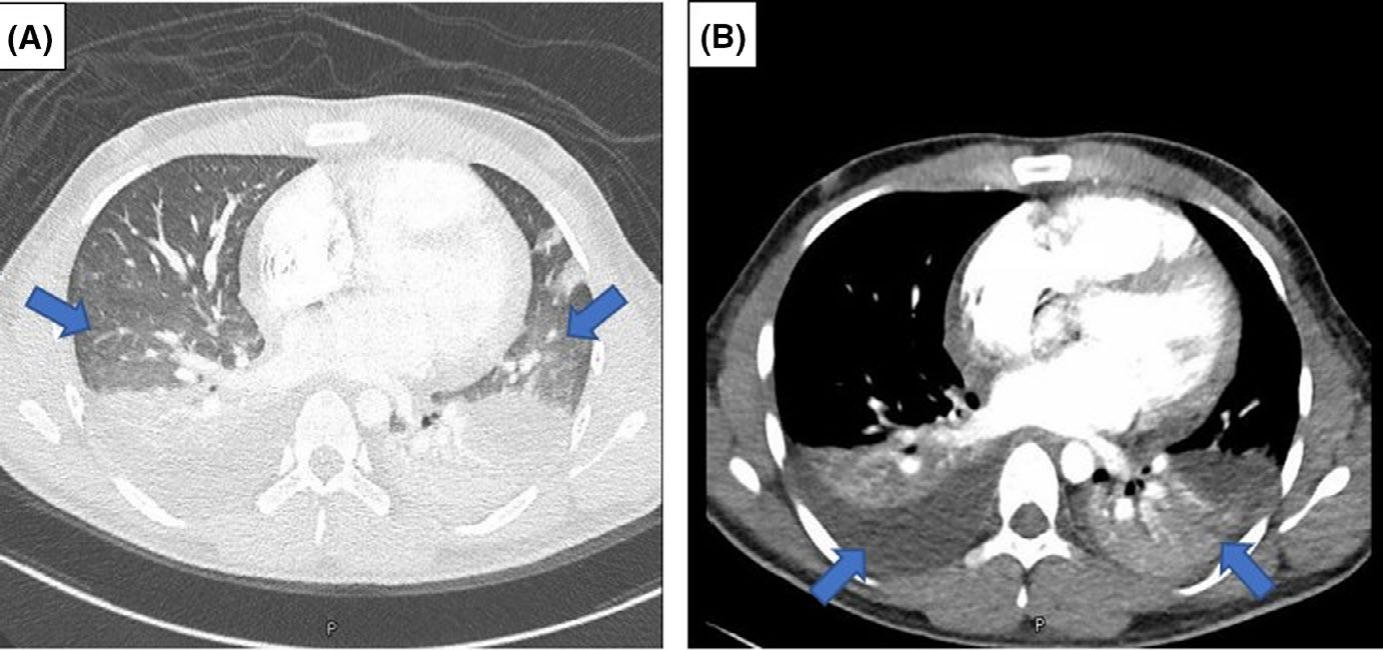
Contrast‐enhanced CT of the thorax. (A) shows bilateral ill‐defined ground‐glass opacities in the lung bases, and (A, B) depicts bilateral pleural effusion and adjacent lung collapse

On the following day of admission, the patient developed respiratory distress and hypotension (Blood pressure was 85/50 mmHg), in addition to the persistent fever and tachycardia. Consequently, the patient was transferred to intensive care unit (ICU) care. Repeated laboratory investigations revealed a drop in hemoglobin level by 2 grams, a decline in platelet count, high D‐dimer and INR, and elevated serum creatinine (Table 1). Repeated ECG and CXR showed sinus tachycardia and new lung infiltrate in the left lower zone, respectively (Figure 1,2). Echocardiography revealed atrial septal defect, moderate pericardial effusion, mild systolic dysfunction (Ejection fraction of 46%), and dilated right ventricle with elevated right ventricular systolic pressure. Echocardiography findings with elevated troponin and N‐terminal pro‐B‐type natriuretic peptide (NT‐proBNP) were suggestive for acute myocarditis. Non‐invasive ventilation was initiated to relieve his respiratory distress. Also, Ceftriaxone was escalated to piperacillin‐tazobactam. Computed tomography (CT) thorax was requested to rule out pulmonary embolism and showed bilateral ground‐glass opacities in the lung bases and bilateral pleural effusion with the adjacent collapse of lower lobes (Figure 3).

On day five of admission, the patient was still febrile and tachypneic. Then, he was intubated to relieve his respiratory distress. All blood cultures did not reveal any organism. We escalated the antibiotic to meropenem and linezolid based on our infectious diseases (ID)team recommendation. After two days of antibiotics escalation, the fever resolved, and the patient started to improve clinically and laboratory‐wise. On day seven of admission, he was extubated successfully and kept on a nasal cannula. Over the next few days after extubation, he remained vitally stable and on room air. Accordingly, the patient was discharged home on day ten of the hospital stay. Cardiology outpatient follow‐up was given to repeat echocardiography and for further management of ASD.

Interestingly, three days after the discharge, the patient presented to the ED with right‐side facial weakness that was consistent with Bell's palsy. No other focal neurological deficit was detected. Prednisolone and ocular lubricant were prescribed as a treatment for Bell's palsy. Currently, the patient is healthy with minimal right facial weakness.

## 3 DISCUSSION

The pathogenesis of MIS in children or adults is still not well described, and multiple pathophysiological mechanisms have been postulated. However, it has been claimed that the dysregulated immune response to SARS‐CoV2 is probably the principal pathophysiology of MIS.^4^ MIS‐A usually develops after two to five weeks of COVID‐19 diagnosis. US centers for disease control (CDC) and the world health organization (WHO) defined the criteria for diagnosing MIS‐C. Both organizations' case definitions require all the following: the presence of fever, features of two or more multisystem involvement, elevated inflammatory markers, evidence of SARS‐CoV‐2 infection (current or previous), or contact with COVID‐19 patients, and finally, the exclusion of other possible diagnoses. WHO included patients between 0 and 19 years, whereas CDC included patients up to 20 years. CDC has been defined as similar for patients aged ≥21 years, known as MIS‐A.^2^, ^5^ Our patient fulfilled MIS criteria. He had a persistent fever; presented with gastrointestinal (abdominal pain, diarrhea, and elevated liver enzymes), hematological(coagulopathy), and cardiac manifestations (myocarditis with elevated troponin and NT‐proBNP); his CRP, ferritin, procalcitonin were elevated; he had a history of COVID‐19 three weeks before presentations; and other possible differential diagnoses, such as sepsis, ruled out.

As aforementioned, MIS is always associated with variable organs dysfunction caused by different mechanisms. In adults with MIS, myocardial dysfunction has been reported in more than 80% of the cases.^3^ Regarding the mechanism of cardiac involvement in MIS, it has been suggested that several causes are responsible for cardiac injury, including direct viral myocarditis, systemic hyper inflammation, and stress‐induced cardiomyopathy.^6^ Incidentally, our patient was found to have ASD with elevated pulmonary artery pressure. In addition, echocardiography revealed global hypokinesia with a decrease in LVEF and pericardial effusion. In addition, NT‐proBNP and troponin levels were significantly elevated (Table 1). All the previous cardiac findings were consistent with acute myocarditis.

Different neurological complications, either central or peripheral, were reported in COVID‐19 patients, and COVID‐19‐related Bell's palsy is one of those complications.^7^, ^8^ Also, Bell's palsy has been reported post‐COVID‐19 infection like in our patient.^9^, ^10^ Bell’s palsy is a lower motor neuron (LMN) facial nerve palsy manifesting with weakness on one side of the face, in the absence of other focal neurological deficits. In a recent retrospective study, the incidence of Bell’s palsy in patients with COVID‐19 was estimated to be 0.08% (approximately 82 per 100,000 cases). Regarding the pathophysiology of COVID‐19 associated Bell’s palsy, it has been postulated that molecular mimicry(between viral and facial nerve antigen ) is the most likely mechanism of this neuropathogenesis.^11^, ^12^The most effective treatment for Bell’s palsy is the corticosteroid therapy, particularly if started within 72 h of symptoms onset. Of note, there is no benefit in adding antiviral medication (like acyclovir) to corticosteroid therapy.^13^Our patient developed Bell’s palsy after few weeks of the diagnosis with COVID‐19, supporting a possible immune‐mediated phenomenon. Also, his facial weakness improved significantly with steroid therapy.

The evidence for the treatment of SARS‐CoV2 associated MIS remains scarce. Guidelines for MIS management in the pediatric population have been published by the American College of Rheumatology (ACR). Intravenous immune globulin (IVIG) is effective in the treatment of moderate‐severe MIS‐C. Pulse methylprednisolone (dose of 2 mg/kg/day) can be used concomitantly with IVIG in severe cases.^14^ Anakinra, canakinumab, and tocilizumab have been used in some cases with MIS‐C, although their roles are still unknown.^15^ The previous treatment modalities have been prescribed for adult patients with MIS and shown to be beneficial in such cases.^3^

In our case, MIS‐A was not suspected because this syndrome was not well‐recognized in the adult population at that time (It was only recognized in the pediatric population). Therefore, our patient did not receive any steroid or IVIG therapies. Despite all cultures being negative, he was treated as a case of sepsis and improved after escalating antibiotics to meropenem and linezolid. Nonetheless, our patient presented with the typical features of MIS‐A (all diagnostic criteria are present), and we believe that his improvement with meropenem and linezolid was just a coincidence with the end of his disease course.

## 4 CONCLUSION

MIS‐A is a serious emerging condition that might follow COVID‐19 infection. The clinical features of this syndrome are non‐specific and can be easily misdiagnosed as sepsis. The management guidelines for MIS‐A are still not well established. Bell’s palsy may also follow COVID‐19 infection, and the pathogenesis is most likely related to the autoimmune phenomenon. Finally, the clinician should put MIS‐A at the top of the differential diagnoses for a patient with febrile illness and multiorgan dysfunction during the next few weeks following COVID‐19 infection.

## CONFLICT OF INTEREST

The authors have no conflict of interest to declare.

## AUTHOR CONTRIBUTIONS

AFA: Literature review and manuscript writing. MSA: Final manuscript review and editing.

## CONSENT

Informed consent was obtained from the patient for the publication of this case report.

## ETHICAL APPROVAL

This case report was approved by the Hamad Medical Corporation’s Medical Research Center (Protocol number: MRC‐04‐21‐419).

## Data Availability

The datasets used and/or analyzed during the current study are available from the corresponding author on request.

## References

[1] Feldstein LR, Rose EB, Horwitz SM, et al. Multisystem inflammatory syndrome in U.S. children and adolescents. N Engl J Med. 2020;383(4):334‐346. 10.1056/NEJMoa2021680w 32598831 PMC7346765

[2] Morris SB, Schwartz NG, Patel P, et al. Case series of multisystem inflammatory syndrome in adults associated with SARS‐CoV‐2 infection — United Kingdom and United States, March–August 2020. MMWR Morb Mortal Wkly Rep. 2020;69(40):1450‐1456. 10.15585/mmwr.mm6940e1 33031361 PMC7561225

[3] Bastug A, Aslaner H, Aybar Bilir Y, et al. Multiple system inflammatory syndrome associated with SARS‐CoV‐2 infection in an adult and an adolescent. Rheumatol Int. 2021;41(5):993‐1008. 10.1007/s00296-021-04843-1 33742229 PMC7978449

[4] Lee PY, Day‐Lewis M, Henderson LA, et al. Distinct clinical and immunological features of SARS‐CoV‐2‐induced multisystem inflammatory syndrome in children. J Clin Invest. 2020;130(11):5942‐5950. 10.1172/JCI141113 32701511 PMC7598077

[5] Li J, Kun T, Mike L. COVID‐19 and multisystem inflammatory syndrome in children and adolescents. Lancet Infect Dis. 2020;20(11):e276‐e288. 10.1016/S1473-3099(20)30651-4 32818434 PMC7431129

[6] Sperotto F, Friedman KG, Son MBF, VanderPluym CJ, Newburger JW, Dionne A. Cardiac manifestations in SARS‐CoV‐2‐associated multisystem inflammatory syndrome in children: a comprehensive review and proposed clinical approach. Eur J Pediatr. 2021;180(2):307‐322. 10.1007/s00431-020-03766-6 32803422 PMC7429125

[7] Montalvan V, Lee J, Bueso T, De Toledo J, Rivas K. Neurological manifestations of COVID‐19 and other coronavirus infections: a systematic review. Clin Neurol Neurosurg. 2020;194:105921. 10.1016/j.clineuro.2020.105921 32422545 PMC7227498

[8] Lima MA, Silva MTT, Soares CN, et al. Peripheral facial nerve palsy associated with COVID‐19. J Neurovirol. 2020;26(6):941‐944. 10.1007/s13365-020-00912-6 33006717 PMC7531061

[9] Tasnim A, Bharta R. A case of multisystem inflammatory syndrome Post‐COVID‐19 infection in an adult. Cureus. 2020;12(2):e11961. 10.7759/cureus.11961 33425537 PMC7788052

[10] Hoffmann DE, Anders MK, Dana C, et al. COVID‐19 myocarditis and postinfection Bell's palsy. BMJ Case Rep. 2021;14(1):e240095. 10.1136/bcr-2020-240095 PMC7802700 33431479

[11] Gaurav N, Holly RJ, Sunder SG, et al. Neurological manifestations of COVID‐19: a systematic review. Crit Care. 2020;24(1):421. 10.1186/s13054-020-03121-z 32660520 PMC7356133

[12] Tamaki A, Cabrera CI, Li S, et al. Incidence of Bell Palsy in patients With COVID‐19. JAMA Otolaryngol Head Neck Surg. 2021;147(8):767‐768. 10.1001/jamaoto.2021.1266 34165518 PMC8227441

[13] Quant EC, Jeste SS, Muni RH, Cape AV, Bhussar MK, Peleg AY. The benefits of steroids versus steroids plus antivirals for treatment of Bell's palsy: a meta‐analysis. BMJ. 2009;339:b3354. 10.1136/bmj.b3354 19736282 PMC2739281

[14] Henderson LA, Canna SW, Friedman KG, et al. American college of rheumatology clinical guidance for multisystem inflammatory syndrome in children associated with SARS‐CoV‐2 and hyperinflammation in pediatric COVID‐19: version 2. Arthritis Rheumatol. 2021;73(4):e13‐e29. 10.1002/art.41616 33277976 PMC8559788

[15] Tang Y, Li W, Baskota M, et al. Multisystem inflammatory syndrome in children during the coronavirus disease 2019 (COVID‐19) pandemic: a systematic review of published case studies. Transl Pediatr. 2021;10(1):121‐135. 10.21037/tp-20-188 33633944 PMC7882293

